# Effects of Methotrexate on Plasma Cytokines and Cardiac Remodeling and Function in Postmyocarditis Rats

**DOI:** 10.1155/2009/389720

**Published:** 2009-10-26

**Authors:** Zhengang Zhang, Pei Zhao, Aihua Li, Xiaolei Lv, Yang Gao, Hongguang Sun, Yongling Ding, Jian Liu

**Affiliations:** ^1^Department of Cardiovascular Disease, The First People's Hospital of Yangzhou, 45 Taizhou Road, Yangzhou, Jiangsu 225001, China; ^2^Division of Internal Medicine, Department of Medicine, Yangzhou University, 11 Huaihai Road, Yangzhou, Jiangsu 225001, China; ^3^Cardiovascular Institute Southeast University at Yangzhou, 45 Taizhou Road, Yangzhou, Jiangsu 225001, China; ^4^Department of Ultrasonography, The First People's Hospital of Yangzhou, 45 Taizhou Road, Yangzhou, Jiangsu 225001, China; ^5^Department of Pathology, The First People's Hospital of Yangzhou, 45 Taizhou Road, Yangzhou, Jiangsu 225001, China; ^6^Central Lab, The First People's Hospital of Yangzhou, 45 Taizhou Road, Yangzhou, Jiangsu 225001, China

## Abstract

Excessive immune activation and inflammatory mediators may play a critical role in the pathogenesis of chronic heart failure. Methotrexate is a commonly used anti-inflammatory and immunosuppressive drug. In this study, we used a rat model of cardiac myosin-induced experimental autoimmune myocarditis to investigate the effects of low-dose methotrexate (0.1 mg/kg/d for 30 d) on the plasma level of cytokines and cardiac remodeling and function. Our study showed that levels of tumor necrosis factor-(TNF-)alpha and interleukin-6 (IL-6) are significantly increased in postmyocarditis rats, compared with the control rats. Methotrexate treatment reduced the plasma levels of TNF-alpha and IL-6 and increased IL-10 level, compared to saline treatment. In addition, postmyocarditis rats showed significant cardiac fibrosis characterized by increased myocardial collagen volume fraction, perivascular collagen area, and the ratio of collagen type I to type III, compared with the control rats. However, MTX treatment not only markedly attenuated cardiac fibrosis, diminished the left ventricular end-diastolic dimension, but also increased the left ventricular ejection fraction and fractional shortening. Collectively, these results suggest that low-dose methotrexate has ability to regulate inflammatory responses and improves cardiac function and hence contributes to prevent the development of postmyocarditis dilated cardiomyopathy.

## 1. Introduction

Congestive heart failure (CHF) is the leading cause of cardiovascular morbidity and mortality. While state-of-the-art therapeutics, including *β*-blockers, angiotensin-converting enzyme inhibitors, and angiotensin-receptor blockers, are available to manage CHF, the prognosis for patients with this condition remains poor [1]. Patients with acute myocarditis generally experience full recovery following treatment for the myocardial inflammation. However, some patients progress to persistent myocardial inflammation and subsequently develop dilated cardiomyopathy (DCM) along with CHF.

Increasing evidence suggests that immune activation and inflammatory mediators may play a role in the development and progression of CHF [2, 3]. For example, elevated plasma concentrations of the proinflammatory cytokines tumor necrosis factor-alpha (TNF-alpha) and interleukin-6 (IL-6) have been frequently observed in patients with CHF [4]. Plasma levels of these cytokines closely correlate with the severity of CHF; they also provide valuable predictive information regarding the patient's prognosis and the rate of new-onset heart failure [5]. Furthermore, elevated TNF-alpha in mice, increased by cardiac restricted over-expression or chronic infusion, facilitates the occurrence of typical phenotypes of CHF [6, 7]. Several studies have shown that patients with some diseases characterized by inflammatory mediator activation (e.g., rheumatoid arthritis, septic shock) are more prone to developing CHF [8]. It is well known that IL-10 is a potent anti-inflammatory cytokine. Intraperitoneal administration of recombinant human IL-10 into mice with experimental viral myocarditis appeared to improve survival, attenuate myocardial inflammation and decrease the myocardial mRNA expression of TNF- alpha, IL-2, and inducible NO synthase [9]. In peripheral blood mononuclear cells from CHF patients, IL-10 reduced lipopolysaccharide-stimulated TNF-alpha production [10]. Moreover, previous study demonstrated level of IL-10 was reduced in patients with CHF [11]. Therefore, it is likely that restoring the balance between pro- and anti-inflammatory cytokines would be a useful treatment of CHF, especially for subjects with proven increased inflammatory activation. Traditional cardiovascular drugs appear to have little effect on the abnormally activated inflammatory mediator network associated with CHF [12]. In contrast, several nonspecific anti-inflammatory treatments and immune-modulating therapeutics have demonstrated favorable effects against CHF [2].

Left ventricular remodeling, as an independent determinant of prognosis and an important therapeutic target in heart failure, is clinically defined as changes in volume, shape, and/or function of the chamber in response to various chronic stresses such as ischemia, pressure/volume overload, and inflammation. High-dose (20–250 mg/kg) methotrexate (MTX) treatment has been indicated for the treatment of neoplastic diseases, such as acute lymphoblastic leukemia and solid cancers, due to its inhibitory effect on *de novo* purine and pyrimidine synthesis through dihydrofolate reductase. In contrast, low-dose MTX treatment has been used as a novel immune modulation therapy [13]. Indeed, MTX has been extensively applied to the management of autoimmune and chronic inflammatory diseases, such as rheumatoid arthritis. MTX is effective against these conditions through its nonspecific modulation of inflammatory mediators [14]. In the present study, we used a rat model of experimental autoimmune myocarditis (EAM) by immunization with cardiac myosin to investigate the effects of low-dose MTX on pro and anticytokine production as well as ventricular remodeling and function.

## 2. Materials and Methods

### 2.1. Reagents

Porcine cardiac myosin and Freund's complete adjuvant were purchased from Sigma (St Louis, MO). MTX (injectable) was obtained from Hualian Pharmaceutical Technology (Shanghai, China). Polyclonal antibodies against collagen I and III and SABC (rabbit IgG)-POD kit were from Wuhanboster Biological Technology (Wuhan, China). Masson's trichrome stain kit was from Fuzhou Dongqi Biological Technology (Fuzhou, China). The ELISA kits used to measure TNF-alpha, IL-6 and IL-10 were from R & D Systems China Co. Ltd (Shanghai, China).

### 2.2. Experimental Autoimmune Myocarditis (EAM) Model

Thirty male, seven-week-old, specific-pathogen-free Lewis rats, weighing 150–200 g, were purchased from the Beijing Vital River Laboratory Animal Technology. A classical model of EAM was induced in 20 of the rats by immunization with porcine cardiac myosin, as previously described with minor modifications [15]. Briefly, porcine cardiac myosin was dissolved in 0.2 M phosphate-buffered saline (PBS) at a concentration of 10 mg/mL and then emulsified with an equal volume of Freund's complete adjuvant supplemented with *Mycobacterium tuberculosis* H37Ra at a concentration of 10 mg/mL. To induce EAM, Lewis rats were subcutaneously injected twice (once on day 0 and once at day 7) in each rear foot pad with 0.1 mL of the emulsified solution. The remaining ten rats were injected with an equal volume of saline in the same manner (control group). All experimental and control animals received humane care and were maintained in the institute's animal facilities under a controlled temperature (22–26°C) and humidity (50%–60%) and given free access to water and standard rat chow. All experimental procedures and protocols were approved by the Special Committee on Animal Welfare of Yangzhou University. The guidelines set by this committee conform to the Guide for the Care and Use of Laboratory Animals published by the US National Institutes of Health (NIH Publication Number 85-23, revised 1996).

### 2.3. Treatment Protocol

Thirty days after immunization with cardiac myosin, the rats (EAM30 group, *n* = 20) were randomly assigned to receive an intraperitoneal injection of either saline (EAM60 group, *n* = 10) or 0.1 mg/kg MTX (EAM-MTX group, *n* = 10), each day for 30 days. Additionally, the control group was injected with saline (*n* = 10) daily for 30 days. The intraperitoneal LD50 of MTX for rats is 6–25 mg/kg [16]. In the present study, we defined the low-dose MTX protocol as daily injections of 0.1 mg/kg, based upon data from a preliminary study.

### 2.4. Echocardiographic Studies

Echocardiographic studies were performed on days 30 and 60 after immunization with cardiac myosin. Rats were weighed and anesthetized by intraperitoneal injection with a mixture of ketamine (25 mg/kg) and diazepam (5 mg/kg). The left ventricular remodeling and function were assessed by transthoracic echocardiography using an 8–11 MHz phased-array transducer (SONOS 7500, Philips Medical Systems). A 2D targeted M-mode echocardiogram was obtained at the level of the chordae tendineae. The following parameters were measured from M- and B-mode tracing: left ventricular end-diastolic dimension (LVEDD, mm), left ventricle end-systolic dimension (LVESD, mm), interventricular septum thickness (IVST, mm), left ventricle posterior wall thickness (LVPWT, mm), left ventricle ejection fraction (LVEF, %), stroke volume (SV, *μ*L), and fractional shortening (FS, %). All parameters were measured using the leading-edge method of the American Society of Echocardiography from three consecutive cardiac cycles, and the average was used for data analysis. Measurements were performed blindly by two independent investigators. Relative wall thickness (RWT) and left ventricular mass index (LVMI, mg/g) were calculated according to following formula [17]: RWT = LVEDD/(IVST + LVPWT), LVM = 0.8[1.04(LVEDD + IVSTd + LVPWTd)^3^ − LVEDD^3^] + 0.6, LVMI = LVM/body weight.

### 2.5. ELISA Detection of Plasma Cytokine Levels

Under anesthesia with a mixture of ketamine and diazepam, blood was drawn from unilatelal eyeball excision on day 30 and from the cardiac ventricular on day 60. All samples were immediately transferred into prechilled tubes containing 75 *μ*L of 10% EDTA-Na_2_. Blood samples were instantly centrifuged at 1000 × g for 20 minutes at 4°C. Plasma was stored at −70°C for later analysis. Concentrations of TNF-alpha, IL-6, and IL-10 were measured in plasma, using commercially available ELISA kits according to the manufacturer's instructions. Each sample was assayed in duplicate and the mean value was calculated. The intraassay coefficients of variance were 3.1%, 7.4%, and 3.4%, respectively. The interassay coefficients of variance were 9.7%, 8.4%, and 8.7%, respectively.

### 2.6. Histopathologic Assessment

On day 60, all rats were bled from cardiac ventricle and then sacrificed under anesthesia. Hearts were removed, rinsed in ice-cold PBS, and transected at the base of the papillary muscles. Transverse sections were fixed with 10% (v/v) phosphate buffered formalin for 12 hours and then embedded in paraffin. Paraffin-embedded tissues were cut into 2 *μ*m thick sections. Sections were stained with hematoxylin-eosin (HE) and Masson's trichrome, according to conventional histological examination techniques. Randomly selected fields (12 per section at a 200 × magnification) were digitized and subject to color threshold analysis. In this analysis, green tissue represents fibrosis while red tissue represents myocardium. Collagen volume fraction (CVF) and perivascular collagen area (PVCA) were measured, based on the green (positive) stained area, which scattered between the surviving myocytes and around the blood vessels, respectively.

Several consecutive sections were used for immunohistochemical staining. Briefly, deparaffinized and hydrated sections were quenched with 3% hydrogen peroxide, digested with compound digestive juice followed by blocking with 5% bovine serum albumin. Tissues were then incubated with the primary antibody, rabbit antirat collagen types I or type III diluted at 1/150, overnight at 4°C. The SABC kit was used for the subsequent steps according to the manufacturer's instructions. Chromogenic development was accomplished using diaminobenzidine-hydrogen peroxide. Slides were then slightly counterstained with hematoxylin and dehydrated, and coverslips were applied. Areas of collagen I and III staining were determined using electronic imaging of the positively stained area, at a 200× magnification. The results are expressed as an area ratio of collagen I to collagen III. The positively stained border zone of vessels was excluded from all calculations. All measurements were quantified by using NIH Image, version 1.30 for Windows.

### 2.7. Statistical Analysis

Data were expressed as mean ± SD. SPSS 12.0 was used for statistical analysis of the data. All data were subjected to one-way ANOVA or *t*-tests. Statistical significance was inferred at *P* < .05 or *P* < .01.

## 3. Results

### 3.1. General Characteristics and Mortality

All rats in the EAM30 group had developed foot ulcers and ankle arthrocele; which were sustained until the end of the study. Prior to being assigned to MTX or saline treatment, the rats which had induced EAM exhibited similar heart rate, blood pressure, and body weight (data not shown). The body weight gain in the control group was higher than in the EAM30 group (368.5 ± 20.5 g versus 307.2 ± 15.8 g). However, the mean body weight of the EAM60 group rats was significantly higher than that of the control group (422.5 ± 17.7 g versus 383.3 ± 18.9 g). Compared with the EAM-60 group, there was a significant decrease of weight gain in the EAM-MTX group (365.7 ± 12.7 g versus 422.5 ± 17.7 g). Also, the foot ulcers and ankle arthrocele were markedly attenuated in the EAM-MTX group. While this study was not designed to statistically evaluate differences between groups in rat survival, it should be noted that no differences in mortality were observed between treatments.

### 3.2. Plasma Cytokine Levels

**Figure 1:**
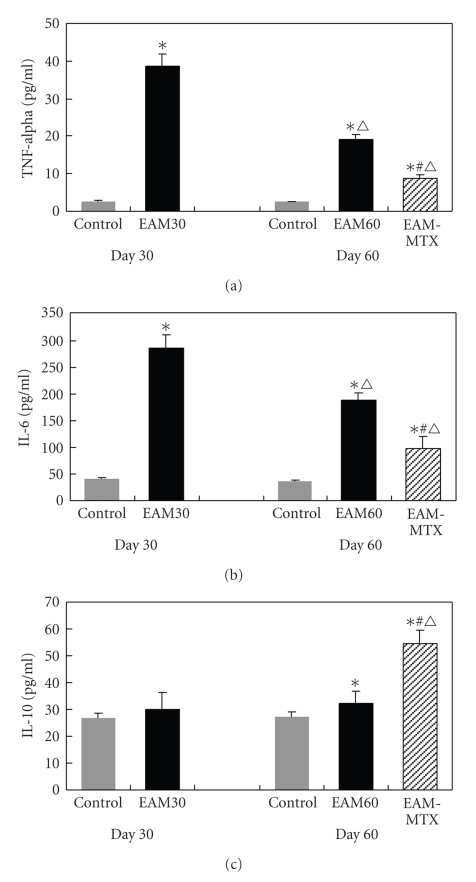
Changes of plasma cytokines levels. The levels of TNF-alpha, IL-6, and IL-10 were determined by ELISA. All results are expressed as mean ± SD (*n* = 10). *P < .01 versus control group; ^*#*^ *P* < .01 versus EAM60 group; ^△^ *P* < .01 versus EAM30 group.

To determine if MTX treatment alters plasma levels of TNF-alpha, IL-6, and IL-10, plasma was isolated and then analyzed by ELISA. As shown in Figure 1, the EAM30 group showed significantly elevated plasma levels of TNF-alpha (14.7-fold) and IL-6 (7.1-fold), compared with the control group (Figures 1(a)  and 1(b)). However, no significant difference in plasma IL-10 levels was observed between the two groups (Figure 1(c)). On day 60, the plasma levels of both TNF-alpha and IL-6 were significantly decreased in the EAM60 group, whereas IL-10 was not changed compared with the EAM30 group. Compared with the EAM60 group, there were significant decreases of the levels of TNF-alpha (−54.8%) and IL-6 (−48.0%) and an increase of IL-10 (68.3%) in the EAM-MTX group. All cytokine levels in the EAM-MTX group were still higher than those in the control group.

### 3.3. Changes in Echocardiographic Parameters

Echocardiographic studies were performed to determine if MTX treatment facilitates the improvement of cardiac structure and function in postmyocarditis rats. Prior to MTX treatment, the parameters of left ventricular remodeling including LVEDD, LVESD, and LVMI in the EAM30 group were higher than the control group (5.43 ± 0.21 mm versus 5.26 ± 0.25 mm, 2.38 ± 0.39 mm versus 2.07 ± 0.32 mm, 1.64 ± 0.14 mg/g versus 1.24 ± 0.12 mg/g, resp.). The cardiac function in the EAM30 group was significantly decreased characterized by reduced FS (46.36% ± 5.69% versus 60.77% ± 5.64%) and LVEF values (68.46% ± 8.13% versus 86.62% ± 5.40%), compared with the control group. There was no statistical difference when comparing any other parameters between the two groups (Table 1). These results suggest that there were significant left ventricular dilation as well as reduced cardiac contractility in postmyocarditis rats.

As shown in Table 2, the EAM60 group has higher LVEDD, LVESD, and RWT and lower LVPWT and IVST than the control group. Moreover, the LVEF, FS, and SV were also all significantly decreased in the EAM60 group compared with the control group, indicating a progressive development of left ventricular remodeling and cardiac dysfunction. In contrast, these were significant decreases of the LVEDD and LVESD and increases of FS, LVEF, and SV in the EAM-MTX group compared to the EAM60 group. The LVMI remained similar among all three groups. These results suggested that MTX treatment was beneficial to reverse the left ventricular dilation and increase cardiac systolic function in postmyocarditis rats.

### 3.4. Histological Study

**Figure 2:**
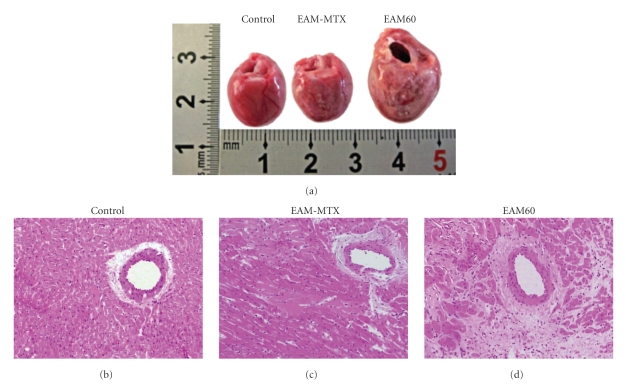
Macroscopic and microscopic assessment of the hearts. (a) Representative gross appearance of the heart from each group of rats. (b) Representative photomicrographs of the ventricular sections (HE staining; ×200). In hearts from the control group, myocytes is well organized. In EAM60 group, the cardiac myocytes exhibited extensive necrosis, degeneration, and disorder. MTX treatment led to a significant decrease in the size of these lesions.

Hearts from the EAM60 group showed macroscopic changes, included severe and diffuse discoloration of the surface tissue. However, the size of the discolored area was significantly reduced in the EAM-MTX group. Additionally, the EAM60 group rats exhibited an enlarged ventricular chamber and thinned wall, as seen in patients with DCM. In contrast, the enlargement of the ventricular chamber was significantly attenuated in the EAM-MTX group (Figure 2(a)). Microscopically, myocardial degeneration, necrotic and alignment disorder of the myocardial fibers, observed in the EAM60 group rats. Interestingly, the extent and area of lesions were dramatically alleviated in the EAM-MTX group (Figures 2(b), 2(c), and 2(d)).

### 3.5. Myocardial Fibrosis

**Figure 3:**
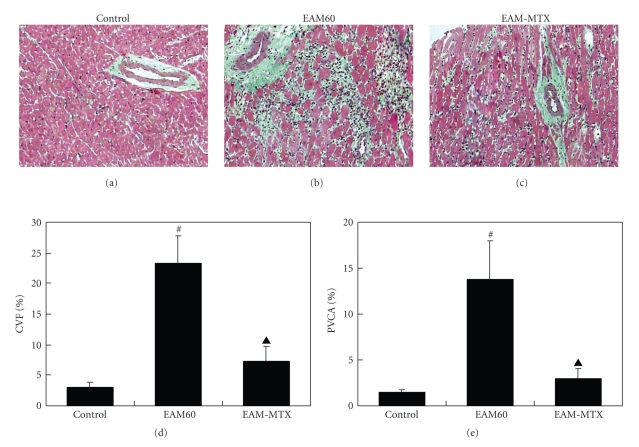
Masson's trichrome staining. Representative photomicrographs of the ventricular sections from the three groups of rats are shown (×200). Green staining represents the collagen fibers (a)–(c). Myocardial collagen volume fraction (CVF; (d)) and perivascular collagen area (PVCA; (e)) were calculated in the control, EAM60 and EAM-MTX groups, respectively. All results are represented as mean ± SD (*n* = 10). *P < .05, ^*#*^ *P* < .01 versus control group; ^▲^ *P * < .01 versus EAM60 group.

Masson's trichrome staining allowed the visualization of any myocardial interstitial fibrosis. As shown in Figure 3(a), only few green staining collagen fibroses were scattered between the red staining myocytes and surrounding the blood vessels in the control group. In contrast, the EAM60 group rats exhibited more extensive and significant green staining of collagen fibers (Figure 3(b)). To quantitatively compare the extent of cardiac fibrosis, CVF and PVCA, were respectively, calculated in this study. The results showed that the EAM60 group rat had higher CVF and PVCA value than the control group (Figures 3(d) and 3(e)), suggesting the occurrence of significant cardiac fibrosis in postmyocarditis. However, in the EAM-MTX group, the green staining of the collagen fibers was markedly reduced, compared with that in the EAM60 group (Figures 3(b)  and 3(c)). Quantitative analysis showed that the CVF and PVCA in the EAM-MTX group were significantly lower than those in the EAM60 group (Figures 3(d)  and 3(e)).

**Figure 4:**
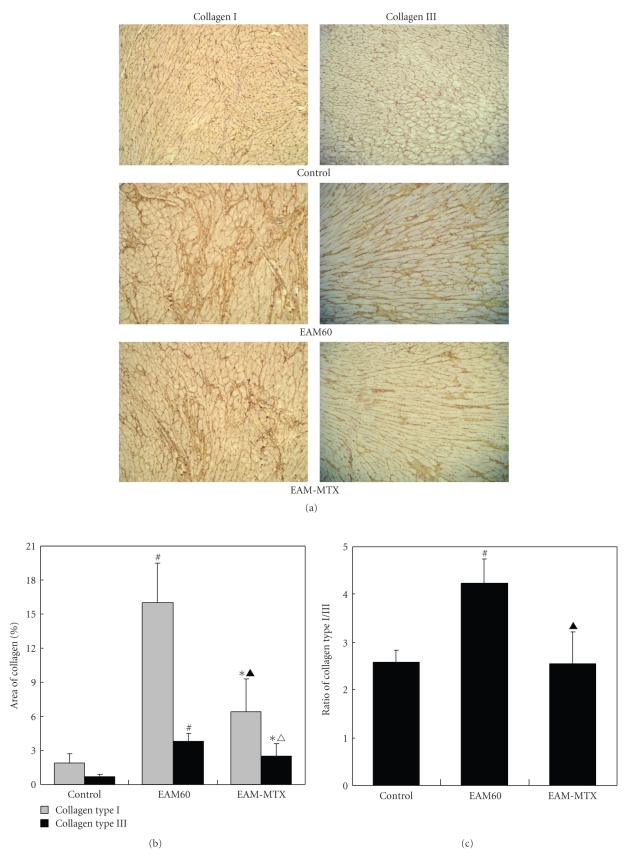
Collagen types I and III staining. Representative photomicrographs of ventricular sections from the three groups of rats are shown ((a); ×200). The areas of collagen types I and III were, measured (b) and the ratios of collagen type I/III were calculated (c). All results are represented as mean ± SD (*n* = 10). *P < .05, ^#^ *P * < .01 versus control group; ^△^ *P * < .05, ^▲^ *P * < .01 versus EAM60 group.

The myocardial collagen matrix, consisting mainly of collagen type I and III, is considered to be an important determinant of myocardial structural integrity and cardiac function. Excessive collagen deposition is essential to the development of cardiac fibrosis. To further identify which kind of collagen deposition mainly occurred postmyocarditis and the role of MTX treatment on them, the collagen type I and III were respectively stained. As a result, the EAM60 group rats showed significantly increased expression of collagen type I and III, mainly type I, in myocardial interstitium, compared with the control group. In the EAM-MTX group, MTX treatment dramatically attenuated the deposition of both collagen types I and III (Figure 4(a)). Quantitative analysis also clearly demonstrated an markedly increase in the size of the area staining for collagen, especially for type I, as well as an increased collagen type I/III ratio in the EAM60 group compared to the control group. Notably, both the staining areas of collagen I/III and collagen type I/III ratio were significantly decreased in the EAM-MTX group compared to the EAM60 group, but still greater than those of the control group (Figures 4(b) and 4(c)).

## 4. Discussion

In the present study, we observed that the proinflammatory cytokines, including TNF-alpha and IL-6, were significantly increased in postmyocarditis rats. However, we demonstrated, for the first time, that MTX administration not only enables to reduce the level of proinflammatory cytokine and increase the level of anti-inflammatory cytokine but also simultaneously attenuates the ventricular dilation and myocardial fibrosis as well as enhances cardiac function.

The EAM model has been extensively characterized in rats and is initiated by immunization with cardiac myosin which, among other effects, provokes a strong inflammatory response. In the recovery phase of the inflammation, immunized animals will progress to DCM [18]. Several studies have demonstrated that early administration of anti-inflammatory therapies can effectively attenuate the induction of EAM [19, 20]. However, it remains unclear whether later anti-inflammatory treatment could further prevent the progression of cardiac remodeling in the stage of postmyocarditis. Therefore, in this study, we used the cardiac myosin-induced rat EAM model to explore the effect of MTX on the development of DCM in postmyocarditis rats.

We recently demonstrated that MTX has significant anti-inflammatory effects for patients with CHF resulting from various causes. Following MTX treatment, these patients showed an improvement in their clinical status [21]. However, MTX treatment failed to improve LVEF and reduce LVEDD in patients with CHF. CHF subjects display a large heterogeneity in the degree of immune and inflammatory activation [2]. Given this, we hypothesized that MTX treatment would be suitable for the treatment of CHF patients, especially for these due to the inflammatory cardiomyopathy. In this study, we showed that MTX treatment not only resulted in significantly decreased plasma levels of TNF-alpha and IL-6 in postmyocarditis rats but more importantly increased the expression of IL-10. Since IL-10, as a potent anti-inflammatory cytokine, is a strong deactivator of monocyte and suppressor of various proinflammatory mediators [9, 10], these results suggest that MTX treatment can obtain a favorable anti-inflammatory net effect. However, it is important to note that we previously failed to observe these effects of MTX in healthy rats (unpublished data). It has been shown that the excessive activation of proinflammatory cytokines TNF-alpha can mimic a number of aspects of the CHF phenotype, including left ventricle remodeling. It is reasonable to propose, then, that the anti-inflammatory effects of MTX may result in improved cardiac remodeling and function in postmyocarditis rats.

Types I and III collagens are major structural proteins forming the myocardial collagen matrix. Excessive collagen deposition in myocardium will lead to cardiac fibrosis. Since Type I collagen determines the stiffness of the myocardium, while type III collagen contributes to its elasticity, the altered collagen I/III ratio has critical impact on the diastolic and systolic function of the heart [22]. In this study, we observed that MTX treatment not only significantly reduced the deposition of collagens but also decreased the collagen type I/III ratio, suggesting a beneficial role of antifibrosis and cardiac function. Meanwhile, we have clearly demonstrated that MTX treatment also markedly relieved ventricular dilation and enhanced cardiac systolic function. Collectively, the above results suggested that low-dose MTX treatment enables to elicit beneficial effects in cardiac remodeling and function in postmyocarditis, maybe through restoring the balance between proinflammatory and anti-inflammatory cytokines. Therefore, in certain extent, it will facilitate to prevent the development of postmyocarditis DCM. Consistent with this study, atorvastatin, an HMG-CoA reductase inhibitor, has recently been shown to regulate the Th1/Th2 cytokine imbalance and to attenuate the histopathological severity of myocarditis [23]. Moreover, in acute myocardial infarction rats, persistent myocardial inflammatory response has adverse effects on LV function and remodeling. However, administration of recombinant IL-10 recently has been shown to significantly suppress infiltration of inflammatory cells and expression of proinflammatory cytokines in the myocardium as well as improve LV function and remodeling by inhibiting cardiac fibrosis [24].

However, the anti-inflammatory mechanism of MTX in EAM rats remains elusive. Previous studies showed that MTX exerts its anti-inflammatory effect by inducing T cell apoptosis, inhibiting neutrophil and mononuclear cells infiltration [17, 25]. However, an in vitro study demonstrated that the anti-inflammatory effect of low-dose MTX is, at least partly, due to regulation of T cell activation and adhesion molecule expression [26]. In addition, in patients with rheumatoid arthritis, MTX treatment has been shown to reduce inflammatory cell numbers as well as levels of monokines and adhesion molecules in the synovial tissue [13].

There are some limitations in this study. Circulating immune cells are the main source of plasma cytokines. To date, the primary source of activated inflammatory mediators involved in CHF remains elusive. Previous studies indicated that autoreactive CD4^+^ T cells and mononuclear cells have a role in the myocardial inflammatory infiltration and the production of cytokines in EAM rats; however, the role of MTX on the function of circulating T cells and mononuclear cells still requires further investigation. Additionally, the regulatory role of MTX on the local, myocardial expression of inflammatory mediators is unclear.

In summary, low-dose MTX treatment facilitates to attenuate the inflammatory response and improve cardiac remodeling and function in postmyocarditis rats.

## Figures and Tables

**Table 1:** Echocardiographic baseline data of control and EAM rats on day 30.

Group	Control (*n* = 10)	EAM30 (*n* = 20)
LVEDD (mm)	5.26 ± 0.25	5.43 ± 0.21*
LVESD (mm)	2.07 ± 0.32	2.38 ± 0.39*
LVPWT (mm)	1.62 ± 0.12	1.55 ± 0.21
IVST (mm)	1.70 ± 0.22	1.58 ± 0.20
LVMI (mg/g)	1.24 ± 0.12	1.64 ± 0.14*
RWT	1.77 ± 0.22	1.80 ± 0.29
FS (%)	60.77 ± 5.64	46.36 ± 5.69*
LVEF (%)	86.62 ± 5.40	68.48 ± 8.13*
SV (*μ*L)	125.64 ± 10.24	132.68 ± 10.69

*P < .05 versus control group.

**Table 2:** Comparison of echocardiograph parameters of all three groups rats on day 60 (*n *= 10).

Groups	Control	EAM60	EAM-MTX
LVEDD (mm)	5.69 ± 0.29	6.46 ± 0.28*	6.06 ± 0.37^△^
LVESD (mm)	2.24 ± 0.30	4.20 ± 0.67^*#*^	2.74 ± 0.41*^,▲^
LVPWT (mm)	1.74 ± 0.17	1.54 ± 0.20*	1.65 ± 0.21
IVST (mm)	1.76 ± 0.17	1.56 ± 0.19*	1.68 ± 0.20
LVMI (mg/g)	1.52 ± 0.04	1.62 ± 0.17	1.60 ± 0.14
RWT	1.73 ± 0.24	2.12 ± 0.32*	1.86 ± 0.35
FS (%)	62.31 ± 6.86	35.27 ± 8.02^*#*^	55.02 ± 4.16*^,▲^
LVEF (%)	89.87 ± 4.40	62.73 ± 10.11^*#*^	84.77 ± 3.60^▲^
SV (*μ*L)	162.38 ± 25.39	132.36 ± 11.30*	156.02 ± 15.29^△^

*P < .05, ^*#*^ *P* < .01 versus control group; ^△^ *P *< .05, ^▲^ *P *< .01 versus EAM60 group.

## References

[1] Mosterd A, Hoes AW (2007). Clinical epidemiology of heart failure. *Heart*.

[2] Gong KZ, Song G, Spiers JP, Kelso EJ, Zhang ZG (2007). Activation of immune and inflammatory systems in chronic heart failure: novel therapeutic approaches. *International Journal of Clinical Practice*.

[3] Jankowska EA, Ponikowski P, Piepoli MF, Banasiak W, Anker SD, Poole-Wilson PA (2006). Autonomic imbalance and immune activation in chronic heart failure-pathophysiological links. *Cardiovascular Research*.

[4] Blum A, Miller H (2001). Pathophysiological role of cytokines in congestive heart failure. *Annual Review of Medicine*.

[5] Valgimigli M, Ceconi C, Malagutti P (2005). Tumor necrosis factor-*α* receptor 1 is a major predictor of mortality and new-onset heart failure in patients with acute myocardial infarction: the cytokine-activation and long-term prognosis in myocardial infarction (C-ALPHA) study. *Circulation*.

[6] Sivasubramanian N, Coker ML, Kurrelmeyer KM (2001). Left ventricular remodeling in transgenic mice with cardiac restricted overexpression of tumor necrosis factor. *Circulation*.

[7] Faircloth M, Clark J, Dighe K, Marber M (2006). The effects of chronic TNF-*α* administration in C57/BL6 mice. *Heart*.

[8] Wolfe F, Michaud K (2004). Heart failure in rheumatoid arthritis: rates, predictors, and the effect of anti-tumor necrosis factor therapy. *The American Journal of Medicine*.

[9] Nishio R, Matsumori A, Shioi T, Ishida H, Sasayama S (1999). Treatment of experimental viral myocarditis with interleukin-10. *Circulation*.

[10] Bolger AP, Sharma R, von Haehling S (2002). Effect of interleukin-10 on the production of tumor necrosis factor-alpha by peripheral blood mononuclear cells from patients with chronic heart failure. *The American Journal of Cardiology*.

[11] Stumpf C, Lehner C, Yilmaz A, Daniel WG, Garlichs CD (2003). Decrease of serum levels of the anti-inflammatory cytokine interleukin-10 in patients with advanced chronic heart failure. *Clinical Science*.

[12] Prabhu SD, Chandrasekar B, Murray DR, Freeman GL (2000). *β*-adrenergic blockade in developing heart failure: effects on myocardial inflammatory cytokines, nitric oxide, and remodeling. *Circulation*.

[13] Dolhain RJEM, Tak PP, Dijkmans BAC, De Kuiper P, Breedveld FC, Miltenburg AMM (1998). Methotrexate reduces inflammatory cell numbers, expression of monokines and of adhesion molecules in synovial tissue of patients with rheumatoid arthritis. *British Journal of Rheumatology*.

[14] Furst DE (1997). The rational use of methotrexate in rheumatoid arthritis and other rheumatic diseases. *British Journal of Rheumatology*.

[15] Yuan Z, Kishimoto C, Shioji K, Nakamura H, Yodoi J, Sasayama S (2002). Temocapril treatment ameliorates autoimmune myocarditis associated with enhanced cardiomyocyte thioredoxin expression. *Cardiovascular Research*.

[16] Genestier L, Paillot R, Fournel S, Ferraro C, Miossec P, Revillard J-P (1998). Immunosuppressive properties of methotrexate: apoptosis and clonal deletion of activated peripheral T cells. *The Journal of Clinical Investigation*.

[17] Devereux RB, Reichek N (1977). Echocardiographic determination of left ventricular mass in man. Anatomic validation of the method. *Circulation*.

[18] Nagaya N, Kangawa K, Itoh T (2005). Transplantation of mesenchymal stem cells improves cardiac function in a rat model of dilated cardiomyopathy. *Circulation*.

[19] Yokoseki O, Suzuki J-I, Kitabayashi H (2001). Cis element decoy against nuclear factor-*κ*B attenuates development of experimental autoimmune myocarditis in rats. *Circulation Research*.

[20] Futamatsu H, Suzuki J, Koga N (2006). A CCR1 antagonist prevents the development of experimental autoimmune myocarditis in association with T cell inactivation. *Journal of Molecular and Cellular Cardiology*.

[21] Gong K, Zhang Z, Sun X (2006). The nonspecific anti-inflammatory therapy with methotrexate for patients with chronic heart failure. *American Heart Journal*.

[22] Pathak M, Sarkar S, Vellaichamy E, Sen S (2001). Role of myocytes in myocardial collagen production. *Hypertension*.

[23] Liu W, Li W-M, Gao C, Sun N-L (2005). Effects of atorvastatin on the Th1/Th2 polarization of ongoing experimental autoimmune myocarditis in Lewis rats. *Journal of Autoimmunity*.

[24] Krishnamurthy P, Rajasingh J, Lambers E, Qin G, Losordo DW, Kishore R (2009). IL-10 inhibits inflammation and attenuates left ventricular remodeling after myocardial infarction via activation of STAT3 and suppression of HuR. *Circulation Research*.

[25] Dalmarco EM, Fröde TS, Medeiros YS (2002). Effects of methotrexate upon inflammatory parameters induced by carrageenan in the mouse model of pleurisy. *Mediators of Inflammation*.

[26] Johnston A, Gudjonsson JE, Sigmundsdottir H, Ludviksson BR, Valdimarsson H (2005). The anti-inflammatory action of methotrexate is not mediated by lymphocyte apoptosis, but by the suppression of activation and adhesion molecules. *Clinical Immunology*.

